# Pediatric siMS score: A new, simple and accurate continuous metabolic syndrome score for everyday use in pediatrics

**DOI:** 10.1371/journal.pone.0189232

**Published:** 2017-12-06

**Authors:** Rade Vukovic, Tatjana Milenkovic, George Stojan, Ana Vukovic, Katarina Mitrovic, Sladjana Todorovic, Ivan Soldatovic

**Affiliations:** 1 Department of Endocrinology, Mother and Child Health Care Institute of Serbia "Dr Vukan Cupic", Belgrade, Serbia; 2 Mother and Child Health Care Institute of Serbia "Dr Vukan Cupic", Belgrade, Serbia; 3 BIDMC – Harvard Medical School, Boston, Massachusetts, United States of America; 4 Department of Pediatric and Preventive Dentistry, School of Dental Medicine, University of Belgrade, Belgrade, Serbia; 5 Institute for Medical Statistics and Informatics, School of Medicine, University of Belgrade, Belgrade, Serbia; Mount Sinai Health System, University of Toronto, CANADA

## Abstract

**Background:**

The dichotomous nature of the current definition of metabolic syndrome (MS) in youth results in loss of information. On the other hand, the calculation of continuous MS scores using standardized residuals in linear regression (Z scores) or factor scores of principal component analysis (PCA) is highly impractical for clinical use. Recently, a novel, easily calculated continuous MS score called siMS score was developed based on the IDF MS criteria for the adult population.

**Objective:**

To develop a Pediatric siMS score (PsiMS), a modified continuous MS score for use in the obese youth, based on the original siMS score, while keeping the score as simple as possible and retaining high correlation with more complex scores.

**Subjects and methods:**

The database consisted of clinical data on 153 obese (BMI ≥95^th^ percentile) children and adolescents. Continuous MS scores were calculated using Z scores and PCA, as well as the original siMS score. Four variants of PsiMS score were developed in accordance with IDF criteria for MS in youth and correlation of these scores with PCA and Z score derived MS continuous scores was assessed.

**Results:**

PsiMS score calculated using formula: (2xWaist/Height) + (Glucose(mmol/l)/5.6) + (triglycerides(mmol/l)/1.7) + (Systolic BP/130)—(HDL(mmol/l)/1.02) showed the highest correlation with most of the complex continuous scores (0.792–0.901). The original siMS score also showed high correlation with continuous MS scores.

**Conclusion:**

PsiMS score represents a practical and accurate score for the evaluation of MS in the obese youth. The original siMS score should be used when evaluating large cohorts consisting of both adults and children.

## Introduction

The pandemic of childhood obesity has resulted in significant concerns regarding associated comorbidities in the pediatric population, with metabolic syndrome (MS) in youth gaining a lot of attention during recent years [1–3]. In 2007, the International Diabetes Federation (IDF) has published the first international definition of MS in children and adolescents [2]. This was an important step which overcame the use of multiple definitions of childhood MS with widely varying criteria [3, 4]. However, some important issues in the evaluation of MS still remain.

First, and most importantly, the dichotomous (present/absent) nature of the current MS definition results in loss of information [5]. Namely, minimal changes of MS parameters in one subject, or clinically negligible differences between two subjects could result in classifying patients as having MS or not. Secondly, the dichotomous nature of MS definition is very limited in quantifying the effects of MS treatment over time [5]. For example, the treatment effect can be quantified only by determining if the patient has or does not have MS after treatment. On the other hand, significant improvements of cardiometabolic risk parameters in a patient which still has MS are undetected by the dichotomous definition.

In order to overcome these limitations of the dichotomous MS definition, an alternative approach to the assessment of MS was developed during the last decade. Several continuous MS scores were developed for both adult and pediatric populations, using standardized residuals in linear regression (Z scores) or factor scores of principal component analysis [6–20]. Although these scoring methods overcame limitations of the dichotomous MS definition, calculation of scores requires the use of sophisticated statistical software and the scores are sample specific [5]. In practical terms, changes in the continuous MS score of a single patient over time could not be evaluated, and neither could differences between patients from different populations (samples). Most importantly, these scores are highly impractical for clinical use.

Recently, a novel continuous MS score called siMS score was developed in order to overcome the shortcomings of the previous MS scores [5]. The siMS score is easily calculated, without the need of a sample database and shows a high correlation with previous complex MS scores. The siMS score was validated in a population which consisted mainly of obese adults, and the score formula is based on the IDF MS criteria for the adult population ^5^. Having in mind the significant differences in the IDF definition of MS in adults vs. children (use of waist percentiles, HDL cutoff), the siMS score should be modified and validated for use in evaluation of the obese youth [2].

The main objective of this study was to develop a Pediatric siMS score (PsiMS), a modified continuous MS score for use in obese children and adolescents, based on the original siMS score. The goal was to derive the PsiMS formula in accordance with the current IDF definition of pediatric MS, while both keeping the score as simple as possible and retaining high correlation with more complex scores. The secondary objective was to validate the original siMS score, and to compare its accuracy with the Pediatric siMS score in evaluation of MS in the obese youth.

## Subjects and methods

### Subjects

We collected clinical data on 153 obese (BMI ≥95^th^ percentile) children and adolescents (88 female and 65 male subjects) who were consecutively referred for evaluation of diet-induced obesity from primary care physicians to the Department of Endocrinology at Mother and Child Health Care Institute of Serbia. Patients with secondary obesity syndromes and other illnesses, as well as patients taking medications known to alter blood pressure, glucose or lipid metabolism were excluded from this study. Written informed consents were obtained from the parents or guardians of all participants and from the patients older than 15 years for admission to Hospital and the procedures performed during the course of standard endocrine workup of obesity in accordance with the Hospital policies. The data were retrospectively collected and the Ethical Committee of Mother and Child Health Care Institute of Serbia granted approval for the present study and waiver for individual written consent on the basis of nonidentifiable use of previously obtained retrospectively collected data. Authors signed written obligation to use these data according to all applicable ethical standards without disclosing the identity of the subjects. The study protocol was formally approved by the Hospital Ethics Committee and in accordance with the Declaration of Helsinki.

### Clinical and laboratory data

Methodology used was similar to our previous research of metabolic comorbidities in the obese youth [21, 22]. BMI percentiles and standard deviation scores (SDS) were calculated in accordance with WHO growth reference charts using WHO Anthro and AnthroPlus software and waist circumference percentiles (WC) according to the reference values by Fernandez et. al [23–25].

Fasting levels of glucose, insulin, triglycerides and HDL cholesterol were measured, and the degree of insulin resistance was determined by the homeostatic model assessment of insulin resistance (HOMA-IR) index, calculated as the product of the fasting glucose and insulin concentrations (in mmol/l and μIU/ml, respectively) divided by 22.5 [26].

### Metabolic syndrome definition and calculation of continuous MS scores

IDF criteria for MS in children and adolescents were used in the development of PsiMS score variants [2]. According to IDF definition, subjects aged <16 years are diagnosed with MS in the presence of abdominal obesity (WC ≥90^th^ percentile for age, or adult cutoff if lower) plus the presence of two or more of the other components: 1. triglycerides ≥1.7 mmol/l, 2. HDL cholesterol <1.03 mmol/l, 3. systolic blood pressure ≥130 mmHg or diastolic blood pressure ≥85 mmHg, and 4. fasting glucose ≥5.6 mmol/l. Adolescents aged ≥16 years were diagnosed with MS in the presence of abdominal obesity (WC ≥94 cm in males and ≥80 cm in females) plus the presence of two or more of the other components: 1. triglycerides ≥1.7 mmol/l, 2. HDL cholesterol <1.03 mmol/l in males and <1.29 in females, 3. systolic blood pressure ≥130 mmHg or diastolic blood pressure ≥85 mmHg, and 4. fasting glucose ≥5.6 mmol/l.

Original siMS score was calculated in all subjects, as well as proposed Pediatric siMS score variants. The original siMS score was calculated according to the following formula [5]:

$$siMS\;score=\frac{2\;x\;Waist}{Height}+\frac{Gly(mmol/l)}{5.6}+\frac{Tg(mmol/l)}{1.7}+\frac{TA\;systolic}{130}-\frac{HDL(mmol/l)}{1.02\;or\;1.28(male/female)}$$

(waist—waist circumference in cm, Gly—glucose, Tg—triglycerides, TA systolic—systolic arterial blood pressure, HDL—HDL cholesterol).

Several Pediatric siMS score (PsiMS) variants based on the original siMS score were also calculated. The PsiMS formulas were calculated as modifications of the original siMS score in accordance with the current IDF definition of pediatric MS, while keeping the score as simple as possible. In the first variant of PsiMS formula (PsiMS v1), HDL cut-off of 1.02 mmol/l was applied to both genders, having in mind that same cut-off (<1.03 mmol/l) is used for children aged less than 16 years in the IDF definition of MS in youth for both genders [2]. In the second variant of PsiMS formula, according to the IDF definition of pediatric MS, waist percentiles were used. The third variant (PsiMS v3) included both the modification in HDL cut-off and waist percentiles. Lastly, although HOMA-IR is not included in the IDF MS definition, having in mind that insulin resistance is considered to play a significant role in the development of MS and is often used for the calculation of continuous MS scores, fourth variant (PsiMS v4) was also included in the analysis, with HOMA-IR instead of fasting glucose. Used PsiMS score variants included following modifications to the original siMS score:

1. PsiMS v1
  $$\frac{HDL\;(mmol/l)}{1.02\;or\;1.28\;(male/female)}\;is\;replaced\;with\;\frac{HDL\;(mmol/l)}{1.02}$$
2. PsiMS v2
  $$\frac{2\;x\;Waist}{Height}\;is\;replaced\;with\;\frac{Waist\;percentile}{90}$$
3. PsiMS v3
  $$\frac{HDL\;(mmol/l)}{1.02\;or\;1.28\;(male/female)}\;is\;replaced\;with\;\frac{HDL\;(mmol/l)}{1.02}$$
  and
  $$\frac{2\;x\;Waist}{Height}\;is\;replaced\;with\;\frac{Waist\;percentile}{90}$$
4. PsiMS v4
  $$\frac{Gly\;(mmol/l)}{5.6}\;is\;replaced\;with\;\frac{HOMA-IR}{2.85}$$

In order to test the PsiMS score validity, several complex continuous MS scores were calculated using sum of Z scores or factor scores of principal component analysis. Four different MS continuous scores were calculated using sum of Z scores [7, 20]. Z scores were calculated by regressing each component of continuous MS score with age and gender. Sum of all Z scores represented continuous MS score. First variant was sum of Z scores, calculated using waist circumference, systolic arterial blood pressure, triglycerides, HDL and glucose regressed for age and gender. Second variant was score calculated using HOMA-IR instead of glucose. Third variant was calculated using waist percentiles, and fourth variant used waist percentiles and HOMA-IR. Triglycerides and HOMA were transformed using logarithmic transformation in order to obtain normal distribution. Z score for HDL was multiplied by -1. Scores derived from principal components analysis (PCA) were calculated in eight different ways, four with weighted sums (weighted for variance explained) of factor scores and four using first component of PCA [6, 27, 28]. Variables and variants used in PCA were identical as in sums of Z scores.

### Statistics

Data are presented as count (%) or mean (standard deviation). Pearson correlation analysis was used to assess significant correlations between different scores. All statistical analyses were performed in SPSS version 20 (SPSS Inc, Chicago, IL) statistical package.

## Results

Study included 153 obese children and adolescents, aged 4.9–18.9 years (12.9±3.2). Among subjects, there were 65 males (42.5%) and 88 females (57.5%). Average body mass index expressed in standard deviation scores (SDS) was 3.17±1.01 SDS (3.04±1.0 in females, 3.34±1.03 in males) and average waist circumference was at 91.4±4.1 percentile (90.7±4.9 in females, 92.2±2.7 in males), that is 97.3±13.4 cm (95.6±14.1 in females, 99.8±11.9 in males).

Correlations between PsiMS score variants and scores calculated as sums of Z scores factors and weighted sum of factors derived from principal component analysis are presented in Table 1.

**Table 1:** Correlation analysis of PsiMS score variants with sums of Z scores of factors and weighted sum of factors derived from principal component analysis*.

	Score				
	Original siMS	PsiMS v1	PsiMS v2	PsiMS v3	PsiMS v4
Sum of Z scores (Gly)	.790	.800	.756	.769	.706
Sum of Z scores (HOMA-IR)	.782	.792	.745	.757	.792
Sum of Z scores (Gly) with waist percentiles	.785	.794	.766	.777	.692
Sum of Z scores (HOMA-IR) with waist percentiles	.785	.794	.761	.771	.787
First component PCA (Gly)	.880	.890	.878	.889	.651
PCA (Gly)	.869	.893	.837	.863	.709
First component PCA (HOMA-IR)	.863	.867	.859	.864	.764
PCA (HOMA-IR)	.878	.891	.849	.864	.839
First component PCA (Gly) with waist percentiles	.892	.901	.904	.913	.655
PCA (Gly) with waist percentiles	.865	.892	.847	.876	.723
First component PCA (HOMA-IR) with waist percentiles	.885	.889	.887	.892	.795
PCA (HOMA-IR) with waist percentiles	.855	.869	.831	.847	.856

*all p values are <0.001; PCA—Principal Component Analysis; Each sum of Z scores was calculated as a sum of Z scores of each metabolic syndrome component regressed for age and gender, using either glucose or HOMA-IR as one of the components.

$$Original\;siMS-siMS\;score\;=\;\frac{2\;x\;Waist}{Height}+\;\frac{Gly}{5.6}+\;\frac{Tg}{1.7}+\frac{TA\;systolic}{130}-\frac{HDL}{1.02\;or\;1.28\;(male/female)}$$

$$PsiMS\;v1-PsiMS\;variant\;1\;=\;\frac{2\;x\;Waist}{Height}+\;\frac{Gly}{5.6}+\;\frac{Tg}{1.7}+\frac{TA\;systolic}{130}-\frac{HDL}{1.02}$$

$$PsiMS\;v2-PsiMS\;variant\;2\;=\;\frac{Waist\;percentile}{90}+\;\frac{Gly}{5.6}+\;\frac{Tg}{1.7}+\frac{TA\;systolic}{130}-\frac{HDL}{1.02\;or\;1.28\;(male/female)}$$

$$PsiMS\;v3-PsiMS\;variant\;3\;=\;\frac{Waist\;percentile}{90}+\;\frac{Gly}{5.6}+\;\frac{Tg}{1.7}+\frac{TA\;systolic}{130}-\frac{HDL}{1.02}$$

$$PsiMS\;v4-PsiMS\;variant\;4\;=\;\frac{2\;x\;Waist}{Height}+\;\frac{HOMA\;IR}{2.85}+\;\frac{Tg}{1.7}+\frac{TA\;systolic}{130}-\frac{HDL}{1.02\;or\;1.28\;(male/female)}$$

**Fig 1:**
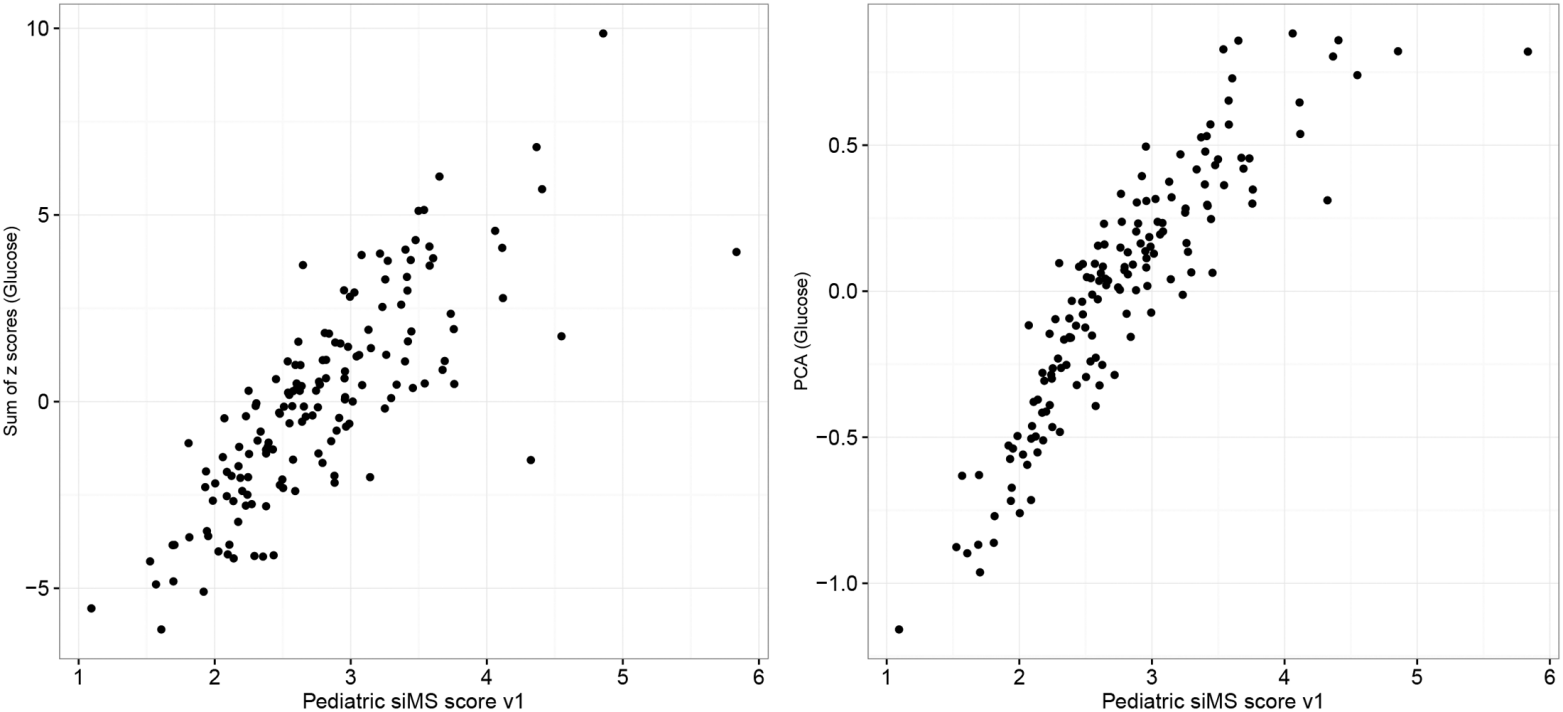
Correlation of pediatric siMS score variant 1 (PsiMS v1) with continuous MS scores calculated using sum of Z scores (with glucose as one of the five factors) and PCA analysis (with glucose as one of the five factors)*. * Sum of z scores (Glucose)–sum of z scores with glucose as one of the five components; PCA (Glucose)–Principal component analysis with glucose as one of five variables.

As shown in Table 1, correlation coefficients of tested siMS score variants with sum of Z scores and PCA analysis were high, with PsiMS variant 1 (HDL cut-off 1.02 mmol/l for both genders) showing the highest correlation with most of the complex continuous scores. The mean PsiMS v1 score was 2.78±0.70 in the whole group, 2.79±0.63 in male and 2.77±0.76 in female subjects. The correlation of PsiMS v1 with sum of Z scores and PCA analysis derived continuous MS scores is graphically presented in Fig 1.

## Discussion

The purpose of this study was to develop a new continuous MS score for evaluation of MS in the obese youth, which would be accurate, easy to calculate and comparable across different populations and studies. Development of such a score would result in overcoming both limitations of the dichotomous IDF MS definition and the impractical aspects of the previous MS scores. In order to accomplish these goals, the PsiMS score was derived from the original siMS score in accordance with the current pediatric IDF MS definition, while both keeping the score as simple as possible and retaining high correlation with more complex scores [2, 5].

Having in mind that the score formula simplicity and its high accuracy are the main factors determining PsiMS future use in research and everyday clinical practice, several variants of PsiMS score were evaluated. Doing so, we analyzed if the simplest formulas had accuracy comparable with more demanding PsiMS variants. The result showed that PsiMS variant 1 (HDL cutoff 1.02 mmol/l for both genders) showed highest correlation with most of the complex continuous scores. This variant is also the simplest to calculate, making it most suitable for everyday clinical practice. The other variants also showed high correlation with complex continuous MS scores, but are more complex for calculation, demanding either calculation of waist percentiles or HOMA-IR. Thus, PsiMS score variant 1 provides an excellent combination of simplicity and high accuracy, and we recommend the use of the following PsiMS formula for calculation of the continuous MS score in the obese youth:

$$PsiMS\;score\;=\;\frac{2\;x\;Waist}{Height}+\;\frac{Gly\;(mmol/l)}{5.6}+\;\frac{Tg\;(mmol/l)}{1.7}+\frac{TA\;systolic}{130}-\frac{HDL\;(mmol/l)}{1.02}$$

It should also be noted, that the original siMS score was also validated in this study and showed high correlation with continuous MS scores. Having in mind that the original siMS score was developed for obese adults, we suggest using the original siMS score when evaluating large cohorts consisting of both adults and children. However, the PsiMS score showed higher correlation with all complex continuous MS scores compared to the original siMS score, and is also simpler to calculate. Thus, we recommend the use of PsiMS score in the evaluation of the obese youth due to its superior accuracy and simplicity. This is particularly important in the field of pediatric endocrinology, where study subjects are almost exclusively in the pediatric age group.

It is well known that the dichotomous nature of the current MS definition results in the loss of information [5, 29]. In order to address this issue, several continuous MS scores were developed [6–20]. However, these scores suffer from many limitations, being sample specific and complex [5]. Sample specificity results in scores from different studies being difficult or impossible to compare. The only way to compare mean scores between studies is to have similar data distribution and similar measures of central tendency and variability, which is made more difficult by the fact that different variables and statistical procedures are used for calculation of MS scores in different studies [5]. Most importantly, complex formulas requiring advanced statistical software limit their use in everyday clinical practice. The development of PsiMS score presented in this study should overcome these issues in the population of the obese youth, since the PsiMS score has high correlation with previous, more complex scores, while being simple and easy to calculate. Also, PsiMS score is not sample specific, which means that scores from different studies can be compared, as well as changes in score of a single patient. It should be noted that PsiMS score was not developed to replace the complex continuous MS scores, but to serve as a surrogate score with high correlation, similar to HOMA-IR or Matsuda indices in the evaluation of insulin resistance [26, 30].

The present study should be interpreted in light of its acknowledged limitations. The study group included only obese Caucasian children and adolescents referred by physicians, thus our findings are limited to this population. However, having in mind that the evaluation for the pediatric MS and calculation of the MS score is most frequently performed in obese children and adolescents, the well selected sample of the obese youth strengthens the findings of our study in this population. Our study is also strengthened by demonstrated high correlation of PsiMS score with the results obtained by using the currently best available continuous MS scores in a well defined sample of the obese youth. Further studies validating PsiMS score in populations of different ethnicities are needed.

In conclusion, the PsiMS score has high correlation with the best available and far more complex continuous MS scores. Also, much simpler calculation of PsiMS score makes it applicable for everyday clinical practice and the scores can be compared between different studies and populations. Therefore, PsiMS score represents a practical and accurate score for the evaluation of MS in the obese youth.

## Supporting information

S1 Dataset S1 Dataset.xlsx—Data set supporting information file. (XLSX)

S1 Calculator S1 PsiMS_calculator.xls—PsiMS score calculator in excel spreadsheet. (XLS)
